# Interleukin-10 gene down-expression in circulating mononuclear cells during infusion of drotrecogin-α activated: a pilot study

**DOI:** 10.1186/cc9252

**Published:** 2010-09-08

**Authors:** Thomas Lavaux, Pascal Bilbault, Anne Launoy, Marie-Pierre Gaub, Pierre Oudet, Francis Schneider

**Affiliations:** 1 Department of Biochemistry and Molecular Biology, Hôpital de Hautepierre et Université de Strasbourg, Avenue Molière, 67098 Strasbourg, France; 2 Intensive Care Unit, Hôpital de Hautepierre et Université de Strasbourg, Avenue Molière, 67098 Strasbourg, France; 3 Department of Anesthesiology, Hôpital de Hautepierre et Université de Strasbourg, Avenue Molière, 67098 Strasbourg, France

## Abstract

**Introduction:**

The purpose of this study was to investigate the gene expression of interferon-gamma (IFN-γ), tumor necrosis factor-alpha (TNF-α) and interleukin-10 (IL-10) in circulating mononuclear cells harvested from septic shock patients on drotrecogin-α activated (DAA) in order to determine whether this treatment has any effect on the inflammation phase.

**Methods:**

We conducted a prospective cohort study in two intensive care departments. Blood samples were collected at inclusion (T1) and 36 hours later (T2) to measure plasma cytokines and the changes in intracellular TNF-α, IL-10 and IFN-γ mRNA expressions using the real-time quantitative polymerase chain reaction (RT-qPCR). Thirty-two septic shock patients were included: 16 with DAA at 24 μg/kg/h for 96 hours (DAA+) and 16 control (DAA-) eligible but contraindicated for DAA because of low platelet count.

**Results:**

The basal characteristics were similar in both groups: mortality (50%), plasma cytokine concentrations, and baseline IFN-γ, TNF-α and IL-10 mRNA expressions (DAA+ vs. DAA-). At T2, there was a significant IFN-γ gene down-regulation in DAA+ but not in DAA- patients (-0.34 (-0.62; +1.54) vs. +1.41 (+0.35; +5.87), *P *= 0.008). In survivors, DAA administration was associated with a down-expression of both IFN-γ (-0.65 (-0.93; 0.48) vs. +0.7 (-0.04; +1.26), *P *= 0.01) and IL-10 (-0.78 (-0.92; -0.6) vs. -0.18 (-0.68; +0.46), *P *= 0.038). In the non-survivors, DAA infusion was associated with IL-10 over-expression when compared with survivors (+0.54 (-0.35; +11.52) vs. -0.78 (-0.92; -0.6), *P *< 0.001).

**Conclusions:**

In this study, lack of IL-10 gene down-expression despite a 36-hour infusion of DAA is an ominous sign in septic shock patients suggesting that DAA is not able to reverse the outcome. Our results suggest that DAA can decrease the expression of anti-inflammatory cytokines in septic shock patients. IL-10 or IFN-γ gene down-expression could represent markers of DAA response.

## Introduction

To improve the outcome, continuous infusion of drotrecogin-α activated (DAA) is recommended for over 96 h at a rate of 24 μg/kg/h in septic shock patients as early as possible [1]. This dosage has raised cost/effectiveness concerns. Yet, since DAA has been made commercially available, *in vitro *studies have highlighted many more properties for this molecule and it can no longer be ignored [2]. A convenient biomarker of its *best use *would be welcome to select patients who could truly benefit from this treatment. This test has never been done for several reasons: (1) single and serial measurements of plasma concentrations of inflammation biomarkers are inconsistent and do not reliably predict outcome in septic patients [3], and (2) since the effect of infused DAA probably changes according to the endogenous concentration of activated protein C at any given moment, it is currently difficult to know if patients are responding to this drug and whether DAA dosage should be adapted when treatment is started without monitoring its plasma concentration over 96 h, which is a complicated and expensive procedure.

In addition, there are methodological pitfalls in septic shock trials relating to the diversity of sources of infection and the exact time the infection started. Also, the inflammatory response triggered by pathogen-associated molecular patterns through Toll-like receptors (TLR) activates a fast response by the nuclear factor kB (NF-kB) pathway. Because the cellular signaling of TLRs can be modified by polymorphism [4] and the subunits of NF-kB expression may be affected by DAA [5], participants in DAA clinical trials should have TLRs and NF-kB expressions as similar as possible so that groups can be appropriately compared. Only then will genes targeted by the NF-kB complex be expressed on a sound basis of comparison. Although, conclusions would be more reliable, large clinical trials taking all these parameters into account would be much too expensive.

This study was conducted in real-life conditions of septic shock management to address the following question: does DAA have any tangible effect on the early pro-inflammatory response to septic shock, measured as a change in TNF-alpha (TNF-α), interferon-gamma (INF-γ), and interleukin-10 (IL-10) mRNA expressions in circulating mononuclear cells (CMNC) harvested from patients and true controls? If so, this would provide an early indication of improvement, and perhaps a DAA efficiency biomarker.

## Materials and methods

This study was approved by our institutional review board for human research and informed written consent was obtained from each participant. Patients with inherited or acquired immunodeficiency were excluded.

Over one year, we included 16 consecutive patients with septic shock [6] treated with DAA (DAA+ group) at the standard dosage (24 μg/kg/h for 96 h), resulting in a mean plasma DAA concentration of 45 ng/mL [2]), and 16 patients as a control group fulfilling septic shock criteria and eligible for DAA but contraindicated (platelet count < 30,000/mm^3 ^and/or uncontrolled bleeding).

We defined T1 as the moment within 12 h after fulfilling septic shock criteria and before giving DAA; T2 was set 36 h later. The Simplified Acute Physiology Score II [7], the Logistic Organ Dysfunction score [8] and 28-day mortality were recorded.

Plasma concentrations of IL-1β, IL-2, IL-4, IL-5, IL-6, IL-8, TNF-α, IL-10, IFN-γ and IL-12 were measured (CBA Human inflammation kit, BD Biosciences, San José, CA, USA) and lympho-monocytes subpopulations determined by flow cytometry (Becton Dickinson, Rungis, France).

Aliquots of 20 ml of whole blood were drawn in EDTA and, CMNC were isolated by Ficoll gradient centrifugation (Eurobio, Les Ulis, France) to obtain aliquots of 10^6 ^cells. RNA extraction and reverse transcription and quantitative real-time PCR were performed with a gene reporter (hydroxymethyl-bilane synthase, HMBS) as reported [9]. The primers for NF-kB sub-units (p50, p65 and IkBa) were purchased from Genome Express, Montreuil, France. For cytokines transcriptional expression (TNF-α, IL-10 and IFN-γ) QuantiTect Primer Assays primers (Qiagen, Courtaboeuf, France) were used. Gene expression was assessed using the 2-DDCT method as reported [10].

The TLR-2 (G2408A) and TLR-4 (A12874G and C13174T) polymorphisms were detected using a hybridization probe assay according to the reported method [4].

### Statistical analysis

T1 and T2 gene expressions are expressed according to previous studies [6]. Values are expressed as mean + SD or as median and interquartile range (25 to 75). For cytokines gene expression, results are expressed as fold change calculated as the ratio (T2-T1)/T1. Kinetic data were studied with analysis of variance for repeated measurements (Friedman's two-way analysis). Comparisons of the mean were made by non-parametric tests (Mann-Whitney U-test or Kruskal-Wallis). Association with the genotype was sought with logistic regression. *P *< 0.05 was considered as significant.

## Results

### Clinical and basic biological characteristics

Clinical characteristics of the groups are shown in Table 1. No difference between the DAA+ group and the control group was found concerning SAPS II (respectively 62.7 ± 2.8 vs 61.5 ± 3.7), LOD score (respectively 9.3 ± 0.8 vs 8.4 ± 0.8) and ICU stay (respectively 32.9 ± 8.8 vs 54.2 ± 19.3 days). The source of infection, types of germ and number of septicaemia were statistically non-significant between the two groups (Table 1). Mortality rate was 50% in both groups.

**Table 1:** Patient characteristics

	DAA + (*n *= 16)	DAA - (*n *= 16)	*P*
Age (yr)	67.4 ｱ 4.0	67.9 ｱ 2.7	NS
SAPS II	62.7 ± 2.8	61.5 ± 3.7	NS
LOD score	9.3 ± 0.8	8.4 ± 0.8	NS
Septicaemia (n (%))	9 (56%)	11 (69%)	NS
ICU stay (days)	32.9 ± 8.8	54.2 ± 19.3	NS
Mortality at Day 28 (n (%))	7 (43%)	8 (50%)	NS
**Type of bacterium : n (%)**			
Gram-positive	8 (50%)	6 (37%)	
Gram-negative	5 (31%)	6 (37%)	NS
Others (fungi)	3 (19%)	6 (37%)	
**Source of infection : n (%)**			
Pneumonia	6 (37%)	9 (56%)	
Peritonitis	5 (31%)	2 (12.5%)	NS
Others	5 (31%)	4 (25%)	
**Toll-like receptors genotyping: n**			
Genotype of TLR-2 (Arg753Arg/Arg753Gln/Gln753Gln)	16/0/0	16/0/0	NS
Genotype of TLR-4 (Asp299Asp/Asp299Gly/Gly299Gly)	12/4/0	15/1/0	NS
Genotype of TLR-4 (Thr399Thr/Thr399Ile/Ile399Ile)	12/4/0	15/1/0	NS

Data are means +/- SD. The respective *P *values result from the non-parametric Kruskal-Wallis test, while associations with the genotype were searched with logistic regression. SAPS II, Simplified Acute Physiology Score II on admission; LOD score, Logistic Organ Dysfunction score; TLRs, Toll like receptors; NS, non-significant.

### TLRs polymorphisms

To assess whether TLRs polymorphism had a role in DAA response, we searched for TLR-2 and TLR-4 polymorphisms in the DAA+ and DAA- groups. Results are indicated in Table 1. No significant difference of distribution was found between the DAA group and the control group regarding TLR-2 or TLR-4 polymorphisms. Concerning TLR-4, we observed a similar prevalence of the double mutation Asp299Gly Thr399Ile in both DAA and control groups (Table 1) as previously described [11].

### Lymphocyte subsets

No statistical change was found between the DAA+ or DAA- groups regarding CD4+ or CD8+ T cells subsets. No change of T cell CD4/CD8 ratio was found over time or between groups (data not shown).

### Pro and anti-inflammatory cytokine profiles

To establish Th1/Th2 profiles, several cytokines were measured in serum at T1 and T2: IL-1β, IL-2, IL-4, IL-5, IL-6, IL-8, TNF-α, IL-10, IFN-γ and IL-12. Only IL-1β, IL-6, IL-8, TNF-α, IL-10 and IFN-γ showed values above the detection threshold. No significant change of cytokine profile was found over time between both studied groups and regarding TLR-4 or TLR-2 polymorphisms (Table 2). We did not find any correlation between intracellular mRNA expression and serum levels of TNF-α, IL-10 and IFN-γ.

**Table 2:** Serum cytokines level of DAA+ and control groups

	DAA+ *N *= 16		DAA- *N *= 16		*P*
	T1	T2	T1	T2	
TNF-alpha (pmol/mL)	15.6(13 to 22.4)	6.6(2 to 11.4)	48(29 to 66)	35(21.1 to 85)	NS
IL-1 (pmol/mL)	32.1(17.1 to 54.1)	30.1(16 to 31)	39.6(20.8 to 58.3)	110.3(88.2 to 132.3)	NS
IL-6 (pmol/mL)	824.5(161.5 to 507.5)	140.7(38,8 to 265)	1,885(1,102.1 to 2,500)	533.2(52 to 1,992.8)	NS
IL-8 (pmol/mL)	623(102.5 to 2,348.3)	110(31.6 to 159.8)	402.3(289.1 to 754.1)	103.1(67.3 to 256.4)	NS
IL-10 (pmol/mL)	47(26.2 to 54)	12.4(8.6 to 23.3)	90(44.8 to 1048.8)	26.7(8.8 to 201.1)	NS
IFN-gamma (pmol/mL)	5(3 to 25)	2(2 to 3)	16(5.3 to 24)	2(2 to 3)	NS

Data are expressed as median (IQR 25 to 75). DAA+, septic shock patients treated with DAA. DAA-, septic shock patients contraindicated to rhAPC treatment. T1, time-point before DAA infusion; T2, time-point 36 h after infusion of DAA. The *P-*value, analysis of variance for repeated data (T1 and T2). NS, non-significant.

### Evolution of intracellular mRNA expressions

From T1 to T2, the fold changes of mRNA expressions of p50, p65, and IkBa NF-kB subunits were not significantly different between groups or between survivors and non survivors (data not shown).

**Figure 1:**
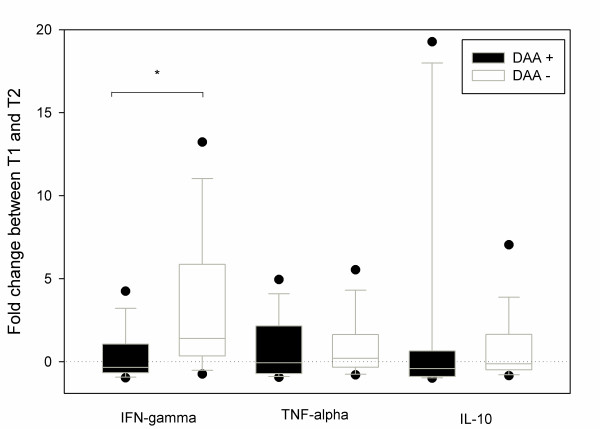
**Evolution of IFN-γ, TNF-α and IL-10 mRNA expressions in CMNCs from DAA + (*n *= 16) DAA- patients (*n *= 16) between inclusion (T1) and after a 36-h DAA infusion (T2)**. Box and whiskers plots show median and interquartile range (25 to 75). Fold change is calculated as the ratio (T2-T1)/T1. DAA: drotrecogin alpha activated. IFN-γ expression is significantly reduced in the DAA+ group (*: *P *= 0.008, Mann-Whitney test) than in the control group.

At T1, IFN-γ, TNF-α and IL-10 mRNA expressions were similar in both groups (DAA+ vs. DAA-). At T2, there was a significant IFN-γ gene down-regulation in DAA+ but not in DAA- patients: -0.34(-0.62; +1.54) vs. +1.41 (+0.35; +5.87) respectively, *P *= 0.008 (Figure 1).

**Figure 2:**
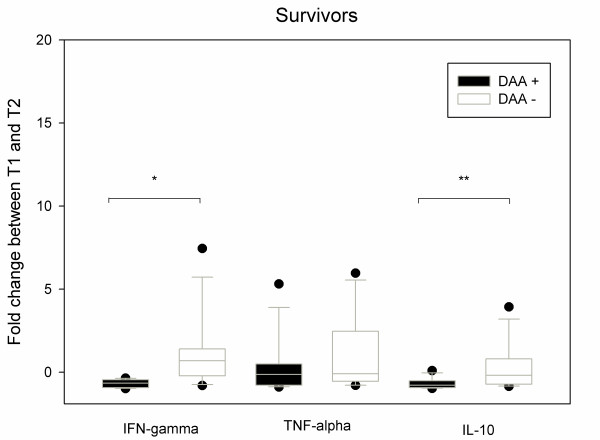
**Evolution of IFN-γ, TNF-α and IL-10 mRNA expressions in CMNCs from DAA + (*n *= 16) and DAA- patients (*n *= 16) between inclusion (T1) and after a 36 h DAA infusion (T2)**. In survivors (*n *= 8) there is a significant lower expression of IFN-γ in the DAA+ group (*: *P *= 0.01, Mann-Whitney test) and also a significant decrease of IL-10 expression in survivors (DAA+ group) than in survivors of controls DAA- (**: *P *= 0.038, Mann-Whitney test). Box and whiskers plots show median and inter quartile range (25 to 75). Fold change is calculated as the ratio (T2-T1)/T1. DAA: drotrecogin-α activated.

**Figure 3:**
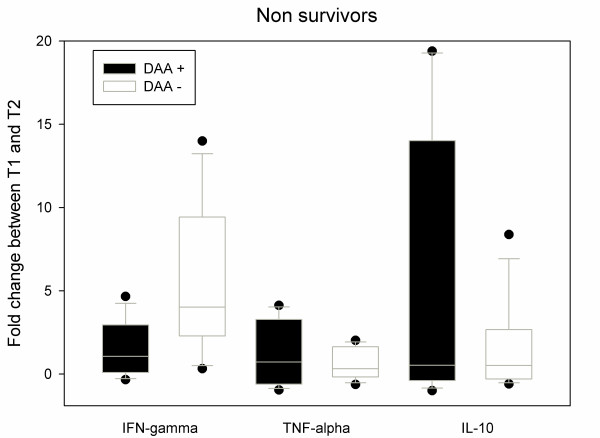
**Evolution of IFN-γ, TNF-α and IL-10 mRNA expressions in CMNCs from DAA + (*n *= 16) and DAA- patients (*n *= 16) between inclusion (T1) and after a 36-h DAA infusion (T2)**. In non-survivor groups (*n *= 8) there is no significant difference of cytokines expression between DAA+ and control groups. Box and whiskers plots show median and inter quartile range (25 to 75). Fold change is calculated as the ratio (T2-T1)/T1. DAA: drotrecogin-α activated.

**Figure 4:**
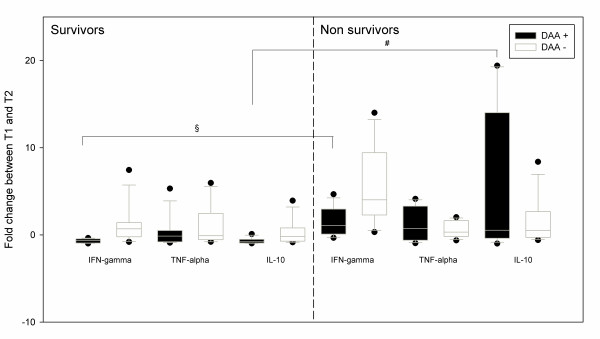
**Evolution of IFN-γ, TNF-α and IL-10 mRNA expressions in CMNCs from DAA + (*n *= 16) and DAA- patients (*n *= 16) between inclusion (T1) and after a 36-h DAA infusion (T2)**. When we compare survivors versus non survivors, there is a significant over-expression for both IFN-γ and IL-10 in DAA+ non survivors compared to DAA+ survivors (§: *P *= 0.038, #: *P *< 0.001 respectively, Mann-Whitney test). Box and whiskers plots show median and inter quartile range (25 to 75). Fold change is calculated as the ratio (T2-T1)/T1. DAA: drotrecogin-α activated.

In the survivors, DAA administration was associated with a down-expression of both IFN-γ (-0.65 (-0.93; -0.48) vs. + 0.7 (-0.04; +1.26), *P *= 0.01) and IL-10 (-0.78 (-0.92; -0.6) vs. -0.18 (-0.68; +0.46), *P *= 0.038) (Figure 2). In non-survivors, there was no significant difference of expression for the three cytokines expression with or without DAA (Figure 3). Nevertheless, in the non-survivors, DAA infusion was associated with IL-10 over-expression (+0.54 (-0.35; +11.52) vs. -0.78 (-0.92; -0.6), *P *= 0.038) and IFN-γ over-expression (+1.07 (+0.22; +02.79) vs. -0.65 (-0.14; -0.78), *P *< 0.001) when compared with the survivors (Figure 4).

Finally, there were no differences between the groups for TNF-α gene expression regardless of the outcome.

## Discussion

In our septic shock patients with clinical and biological characteristics as similar as possible to real-life conditions, a continuous infusion of DAA resulted in changes in the IFN-γ gene expression, suggesting that the drug had early anti-inflammatory effects, yet IL-10 was not significantly reduced. Moreover, when survival was considered, IL-10 dampened the pro-inflammatory reaction of early septic shock in the survivors, whereas it failed to do so in the non-survivors. The lack of IL-10 gene expression decrease by CMNC is associated with an ominous outcome even if full supportive treatment is provided. These data suggest that: (1) DAA infusion interferes *in vivo *with IFN-γ and IL-10 gene expressions through NF-kB-independent modulation, (2) continued IL-10 and IFN-γ gene expression despite a 36-h infusion of DAA may indicate that a standard dosage of DAA fails to affect outcome.

Increased plasma concentration of cytokines such as IFN-γ, TNF-α or IL-10 is a hallmark of septic shock and has negative consequences for recovery, although no clinically relevant thresholds have been proposed [3,12]. However, data on plasma cytokine concentrations do not seem to determine outcome because the exact onset of sepsis-driven cytokines release is usually not clearly definable in septic patients [13]. Therefore, in this study we checked cytokine gene expression by CMNC, and confirm that changes to them occur without consequences on plasma levels in accordance with previous studies [14]. The most significant drawbacks of this approach are the routine availability of the RT-qPCR technique in a hospital laboratory and the time required to obtain the lab test result, which nevertheless takes a few hours (less than six hours) with commercially available techniques of molecular biology.

Our data are in partial accordance with *in vitro *studies that claim that DAA up-regulates IL-10 in LPS-stimulated human monocytes [15]. Yet *in vitro *the DAA concentration required is significantly higher (120 ng/mL) than that achieved *in vivo *with standard infusion (45 to 52 ng/mL) [2,16]. In our setting, IL-10 gene expression was dramatically increased in the non-surviving patients given DAA suggesting either a greater IL-10 gene up-regulation by DAA in patients with poor prognosis, or simply that DAA has no effect on high IL-10 up-regulation by sepsis itself. Since the baseline gene expression levels were not significantly different between groups whatever the outcome and treatment, we suggest that DAA has an influence that is not necessarily powerful.

One of the limitations of our study is that it was underpowered but our hypothesis can not be tested in a random fashion in a large population of patients since DAA administration can not be declined in the absence of contraindication for ethical reasons. Furthermore, in septic shock DAA may actually require a higher infusion rate than the standard one to reach cellular efficiency (that is, dampening IL-10 gene expression). Conversely, DAA may have no effect on patients with continuous IL-10 expression. Hence, maintaining DAA for 96 h as recommended would be questionable. In both situations, the level of IL-10 up regulation in CMNC after 36 h DAA infusion may become a biomarker of efficiency. The time required to obtain the IL-10 gene expression ratio is technically feasible during the working hours of the laboratory: consequently, in a patient infused for 36 h with a T2 test indicating a lack of IL-10 and IFN-γ gene down-regulation, the standard DAA dosage could be reduced after approximately half the scheduled dosage has been infused (which would result in a 50% saving of the drug). This warrants confirmation by a prospective randomized placebo study including only patients with IL-10 over expression.

## Conclusions

If IL-10 and/or IFN-γ gene down-expression fail to occur despite 36 h of DAA infusion in septic shock patients, maintaining treatment for 96 h as recommended becomes questionable. IL-10 and IFN-γ gene expressions by CMNC could become a biomarker of DAA efficiency in this setting.

## Key messages

• Drotrecogin-α (activated) has early anti-inflammatory effects at the transcriptional level on circulating mononuclear cells.

• Checking the transcriptome of cytokines/chemokines in immune cells could be a new approach for monitoring the effect of drotrecogin-α (activated) in sepsis.

## Abbreviations

CMNC: circulating mononuclear cells; DAA: drotrecogin-α activated; EDTA: ethylenediaminetetraacetic acid; HMBS: hydroxymethyl-bilane synthase; ICU: intensive care unit; IFN-γ: interferon-gamma; IL-1β-2-4-5-6-8-10-1: interleukin-1beta-2-4-5-6-8-10-12-10; LOD score: logistic organ dysfunction score; mRNA: messenger ribonucleic acid; NF-kB: nuclear factor kappa-light-chain-enhancer of activated B cells; RT-QPCR: real-time quantitative polymerase chain reaction; SAPS II: simplified acute physiology score II; TLR: Toll-like receptors; TNF-α: tumor necrosis factor-alpha

## Competing interests

The authors declare that they have no competing interests.

## Authors' contributions

TL and PB contributed to the conception of the study, and the analysis and interpretation of data. AL contributed to the acquisition of data. PB and FS wrote the manuscript, and MPG revised it for biological content. FS and PO gave final approval.
